# Osteoblast Response to Copper-Doped Microporous Coatings on Titanium for Improved Bone Integration

**DOI:** 10.1186/s11671-021-03602-2

**Published:** 2021-09-20

**Authors:** Lai-jie Wang, Xiao-hui Ni, Fei Zhang, Zhi Peng, Fu-xun Yu, Lei-bing Zhang, Bo Li, Yang Jiao, Yan-kun Li, Bing Yang, Xing-yuan Zhu, Quan-ming Zhao

**Affiliations:** 1 Department of Orthopedics, Huai’an People’s Hospital of Hongze District, Huai’an, 223100 Jiangsu China; 2 Department of Orthopaedics, Guizhou Provincial People’s Hospital, Guiyang, 550002 Guizhou China; 3 Department of Orthopedics, Dafeng People’s Hospital, Yancheng, 224100 Jiangsu China

**Keywords:** Microarc oxidation, Coating, Osteogenesis, Copper, Osseointegration

## Abstract

Due to their excellent mechanical properties and good biocompatibility, titanium alloys have become a popular research topic in the field of medical metal implants. However, the surface of the titanium alloy does not exhibit biological activity, which may cause poor integration between the interface of the titanium implant and the interface of the bone tissue and subsequently may cause the implant to fall off. Therefore, surface biological inertness is one of the problems that titanium alloys must overcome to become an ideal orthopedic implant material. Surface modification can improve the biological properties of titanium, thereby enhancing its osseointegration effect. Copper is an essential trace element for the human body, can promote bone formation and plays an important role in maintaining the physiological structure and function of bone and bone growth and development. In this study, a microporous copper-titanium dioxide coating was prepared on the surface of titanium by microarc oxidation. Based on the evaluation of its surface characteristics, the adhesion, proliferation and differentiation of MC3T3-E1 cells were observed. A titanium rod was implanted into the rabbit femoral condyle, and the integration of the coating and bone tissue was evaluated. Our research results show that the microporous copper-titanium dioxide coating has a nearly three-dimensional porous structure, and copper is incorporated into the coating without changing the structure of the coating. In vitro experiments found that the coating can promote the adhesion, proliferation and differentiation of MC3T3-E1 cells. In vivo experiments further confirmed that the titanium copper-titanium dioxide microporous coating can promote the osseointegration of titanium implants. In conclusion, copper-titanium dioxide microporous coatings can be prepared by microarc oxidation, which can improve the biological activity and biocompatibility of titanium, promote new bone formation and demonstrate good osteoinductive properties. Therefore, the use of this coating in orthopedics has potential clinical application.

## Introduction

A medical hard tissue implant material must have appropriate mechanical properties, such as strength, elastic modulus, wear resistance and fatigue resistance, so that the implant can bear the physiological load of the implanted area for a long time. At the same time, the material must have good biocompatibility and even bioactivity so that the implant can form a good combination with the physiological tissue of the implantation area without causing adverse reactions in the human body. Pure titanium and titanium alloys have good mechanical properties and biocompatibility and are currently the most widely used metal implant materials.

After the implant is implanted, a series of biochemical reactions first occur on the surface of the material, and the surface characteristics play a vital role in the response of the implant to the internal environment. The microstructure and chemical composition of the implant surface can change the adsorption of proteins, and proteins regulate cell adhesion and ultimately determine their function [1]. Although titanium and titanium alloys are the most widely used orthopedic implant materials, titanium has no biological activity. After implantation in the body, it cannot form a chemical bond with the bone tissue of the implantation area. Titanium and titanium alloys mainly rely on mechanical interlocking to achieve retention [2] and are not conducive to long-term physical function in the body.

The surface coating of titanium implants can complement the mechanical properties of titanium and overcome its shortcomings related to poor biological activity; that is, titanium as a substrate can provide mechanical properties, and elements with good surface structure and biological activity are used as the coating. This layer provides biological activity, and research in this area has become a research hotspot.

At present, the technologies used for the preparation of bioactive coatings on metal surfaces mainly include plasma spraying, ion beam-assisted deposition, electrophoretic deposition, pulsed laser physical vapor deposition, microarc oxidation, magnetron sputtering deposition, sol–gel, direct laser cladding and laser ablation [3–10]. Among them, plasma spraying technology has commercial applications; however, the current coating technology still cannot meet clinical requirements, mainly due to the following problems: the bioactive phase of the coating has low crystallinity and poor bioactivity; the bonding strength between the coating and the substrate is not good; the internal material of the coating is easily dissolved, which affects the long-term stability of the coating in the body; and the preparation process for the coating is too complicated, the process conditions are strict, the cost is high, and the efficiency is low [11].

Micro-arc oxidation, an effective surface modification technology that is currently widely used in the surface modification of metal implants, uses the instant sintering effect of plasma high temperature and high pressure; not only does the surface of the material generate a rough and porous surface, but biologically active elements can also be introduced into the coating. The surface of the material modified by micro-arc oxidation technology can significantly improve the surface morphology, roughness, hydrophobicity and surface energy of the matrix material and other physical and chemical properties so that the biological activity and biocompatibility of the material are greatly improved. The osseointegration between the imported material and bone tissue is of great significance [12].

Copper (Cu) is an essential trace element in the human body that has a variety of functions, including promoting the growth of osteoblasts and promoting the expression of vascular endothelial growth factor in intimal tissues, which is beneficial to the adhesion and proliferation of vascular endothelial cells. Cu also enhances the lipid peroxidation reaction, inhibits the synthesis of bacterial active DNA and related enzymes that interfere with bacterial energy metabolism and does not easily lead to drug resistance [13]; more importantly, copper ions in a certain concentration range are recognized as having both high biological activity and excellent antibacterial properties. Therefore, copper ions have been proven to have good biocompatibility when used in the design of biomaterials [14].

The interaction between the cells and the implant is an important factor in inducing the formation of an osseointegration interface. This interaction mostly depends on the material properties of the implant surface, such as the chemical composition of the surface, surface energy, surface charge and surface morphology. This interaction between cells and implants can affect cell adhesion, proliferation and differentiation. A porous surface has been proven to enhance cell adhesion, proliferation and differentiation, and implants doped with inorganic ions (zinc, strontium, magnesium, etc.) have also been proven to promote osseointegration [15, 16].

The process of microarc oxidation equipment is simple, and the properties of the prepared coating can be adjusted. Coatings doped with different ions can be prepared by changing the composition of the electrolyte. Copper plays an important role in the organism. The appropriate amount of copper ions in the coating can promote the proliferation of osteoblasts and inhibit the adhesion of surface bacteria. Therefore, in this study, we prepared a microporous Cu–TiO_2_ coating on the titanium surface and used in vitro cell experiments and animal experiments to observe and analyze the effect of the microporous Cu–TiO_2_ coating on the surface activity and biocompatibility of titanium. We also sought to explore the feasibility of microporous Cu–TiO_2_ coating as a new type of implant coating material to enhance the adhesion, proliferation and osteogenic differentiation of MC3T3-E1 cells and promote the formation and osseointegration of new bone at the implant-bone interface to lay a theoretical and experimental basis for the clinical application of microporous Cu–TiO_2_ coating on the surface of titanium implants.

## Materials and Methods

### Sample Preparation and Characterization

Through wire cutting, the titanium was processed into a sample with a diameter of 12 mm and a thickness of 1 mm. The implants were made into titanium rods with a diameter of 3 mm and a length of 8 mm. Sandpaper was smoothed and washed with acetone and deionized water to prepare the coating. In this study, a homemade low-power microarc oxidation power supply was used; the micro-arc oxidation voltage was 450 V, the mode was constant-current, the microarc oxidation time was 5 min, and the frequency was 1000 Hz.

The blank control group was labeled Ti, and calcium acetate and calcium glycerophosphate were used as the basic electrolyte solutions. The Ti samples after microarc oxidation in the basic electrolyte solution were labeled with TCP, and the samples after microarc oxidation with different copper oxide contents in the basic electrolyte solution were labeled with TCP Cu I and TCP Cu II (Table 1).

**Table 1:** Electrolyte composition of different coatings

Coating	Aqueous electrolyte concentration (*M*)		
	Calcium acetate	Calcium glycerophosphate	Copper acetate
Ti	0.05	0.02	–
TCP	0.05	0.02	–
TCP CuI	0.05	0.02	0.03
TCP CuII	0.05	0.02	0.06

Field emission scanning electron microscopy (FE-SEM) was used to observe the surface morphology of the samples, energy dispersive spectroscopy (EDS) was used to observe the element distribution on the coating surface, and surface roughness profiler was used to evaluate the roughness of different samples. X-ray diffraction (XRD) and X-ray photoelectron spectroscopy (XPS) were used to observe the elemental composition and microstructure of the coating phase and chemical state.

### Cell Culture

MC3T3-E1 cells (extracted from mouse skull cells) were used for in vitro cell testing. The cells were incubated in MEM with 10% fetal bovine serum α at 37 ℃ in 5% CO_2_. When the cells fused and grew to 80% density, they were digested and passaged with 0.25% trypsin. The third passage was used for cell experiments.

### Live/Dead Staining

The cytotoxicity of each group was evaluated by live/dead fluorescence staining. The cell seeding density was 1.5 × 10^4^ cells/cm^2^. After 3 days of culture, the samples were rinsed with sterile PBS and treated according to the live/dead viability/cytotoxicity kit. The cytotoxicity of the material was observed by fluorescence microscopy.

### Cell Adhesion and Proliferation

The cells were inoculated on the surface of each group at a density of 1.5 × 10^4^ cells/cm^2^. After incubating for 1 h, 2 h and 6 h, the cells were rinsed with PBS and fixed with 4% paraformaldehyde for 30 min. After rinsing with PBS, 40 μL of DAPI staining agent was dropped on the surface of the sample for 10 min to avoid light staining, and the samples were observed and imaged with a laser confocal microscope.

The cell seeding density and culture method were the same as above, and the proliferation activity of the cells was measured with the CCK-8 kit 1, 3, and 10 days after cell culture.

### Expression of Genes Related to Osteogenic Differentiation

The cell inoculation density and culture method were the same as above. The cells were collected 1, 3, and 10 days after inoculation, and real-time quantitative PCR was used to detect the mRNA levels of osteogenic differentiation-related genes (including *BMP*, COL-I, *ALP* and *OCN*). The expression levels of the target genes were normalized to those of the housekeeping gene *GAPDH*. The primer sets are listed in Table 2.

**Table 2:** qRT-PCR primers

Gene	Forward primer sequences (5'-3')	Reverse primer sequences (5'-3')
BMP-2	TCGAAATTCCCCGTGACCAG	GGGACCCTTAGGCCATTGTGTA
ALP	TTGGGCAGGCAAGACACA	GAAGGGAAGGGATGGAGGAG
COL-I	GCCTCCCAGAACATCACCTA	GCAGGGACTTCTTGAGGTTG
OCN	ACCATCTTTCTGCTCACTCTGCT	CCTTATTGCCCTCCTGCTTG
GAPDH	TGCTGGTGCTGAGTATGTGGT	AGTCTTCTGGGTGGCAGTGAT

### Animals and Surgery

The animal experiments were approved by Institutional Animal Care and Use Committee of the Guizhou Provincial People's Hospital. Sixteen adult rabbits, both male and female, weighing 3.6 kg (3.2–3.9 kg) were purchased from the Experimental Animal Center of Soochow University and were divided into an experimental group and a control group (8 rabbits each). Intravenous anesthesia was performed with 3% sodium pentobarbital (0.1 mL per kilogram of body weight). After skin preparation, the skin was fixed and routinely disinfected. A longitudinal incision was made in the lateral femoral condyle to expose the lateral condyle. Using a surgical electric drill, a hole with a diameter of 2.7 mm and a depth of 6 mm was drilled on a flat surface. Two sets of titanium rods were implanted into the bone defect (the left in the experimental group, and the right in the control group), and penicillin was given for 3 consecutive days after the operation to prevent infection.

### Micro-CT Assay

After the operation, the rabbits were kept in separate cages, and they were free to eat and drink water. At 4 w and 8 w postoperation, 8 rabbits were euthanized by air embolization. The femoral condyles of the rabbits containing titanium rods in the experimental group and the control group were removed, the sample size was trimmed, the samples were formalin fixed, and micro-CT scanning was used for three-dimensional reconstruction. The region of interest (ROI) was set using Micro-CT software, and the bone volume fraction of the region of interest (bone volume/total volume, BV/TV %) was measured.

### Histological Evaluation by Toluidine Blue and Fuchsin-Methylene Blue Staining

The samples were dehydrated with gradient alcohol (70%, 80%, 85%, 90%, 95%, 100%, 100%). The dehydrated samples were embedded with methyl methacrylate. After successful embedding, the samples were cut and trimmed, fixed on a slicer with adhesive for cutting, and finally polished with sandpaper to a slice thickness of approximately 20–30 μm. (1) Toluidine blue staining: toluidine blue dye was added to the surface of the slice, and the slice was dyed in a water bath. The surface dye was dried with filter paper, rinsed, and dried naturally in air. The film was sealed and observed under a light microscope. (2) Fuchsin-methylene blue staining: the method was the same as toluidine blue staining. First, methylene blue staining solution was added to the surface of the slices for dyeing, dried in air and rinsed. Then, the slices were soaked in fuchsin staining solution for dyeing, dried naturally in air, sealed and observed.

### Statistical analysis

Data are expressed as the means ± standard deviation determined by SPSS 16.0 software. One-way ANOVA and the SNK test were used to compare the differences between groups. *P* < 0.05 indicates a significant difference.

## Results

### The Surface Morphology, Phase and Chemical Element Composition of the Sample

**Fig. 1:**
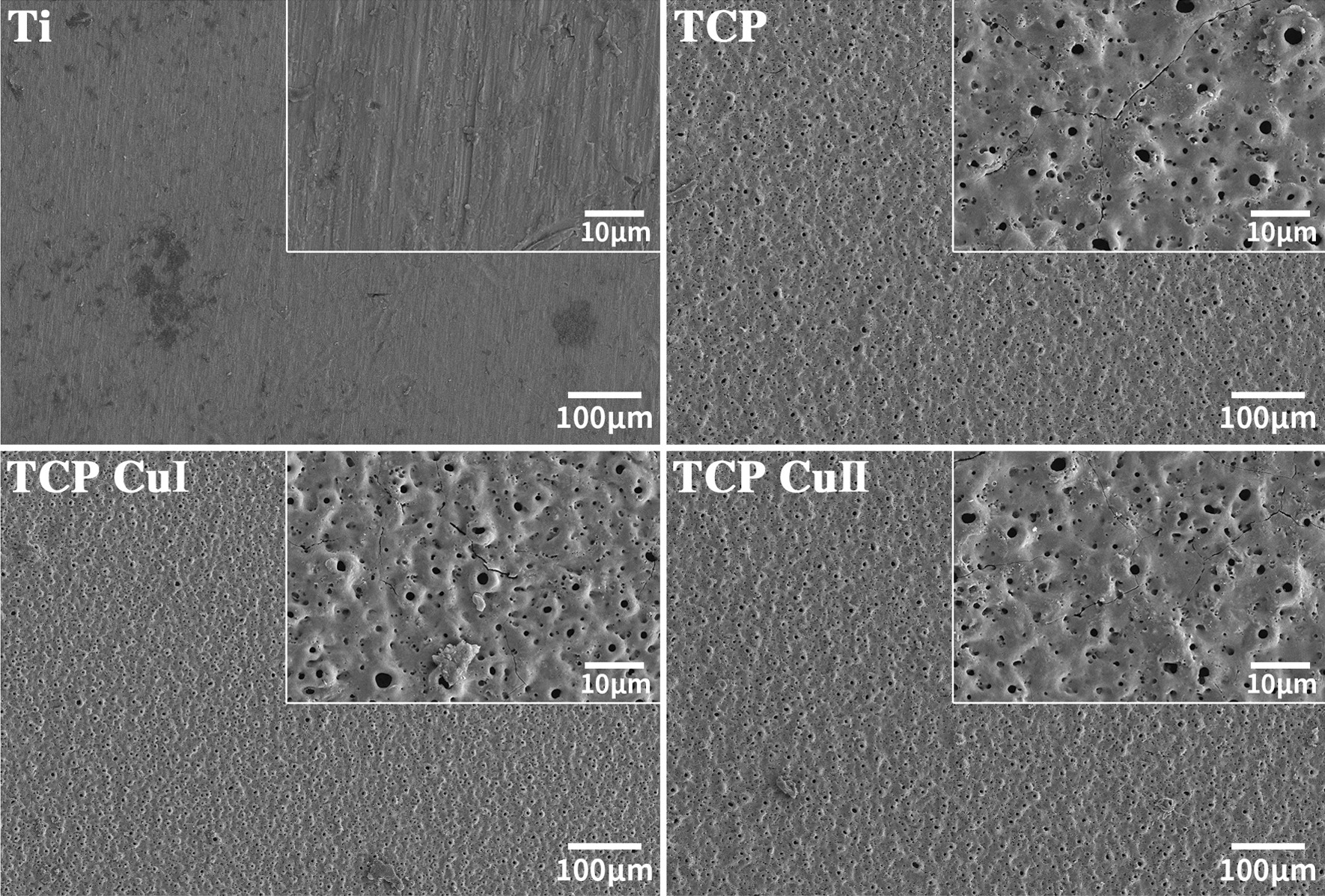
Surface morphologies of Ti, TCP, CP Cu I and TCP Cu II

Figure 1 shows the SEM surface morphologies of different samples. The morphologies of the Ti group and other groups are significantly different. The Ti group has no holes on the surface, and the surface is relatively flat, leaving only some scratches. The TCP group has a typical microarc oxidation surface morphology, and the surface is covered with micropores of different sizes. These micropores intersect each other and have an approximate "three-dimensional structure". Large and small pores are nested with each other, and the pores have no fixed rules. The shape is irregular. In addition, there are burn marks in the gaps between the holes. Similar to the surface morphology of the TCP group, the surfaces of the TCP Cu I and TCP Cu II groups are also covered by irregularly shaped micropores, and there is no obvious difference in surface morphology between the groups. The doping of copper does not affect the structure and morphology of the micropores.

**Fig. 2:**
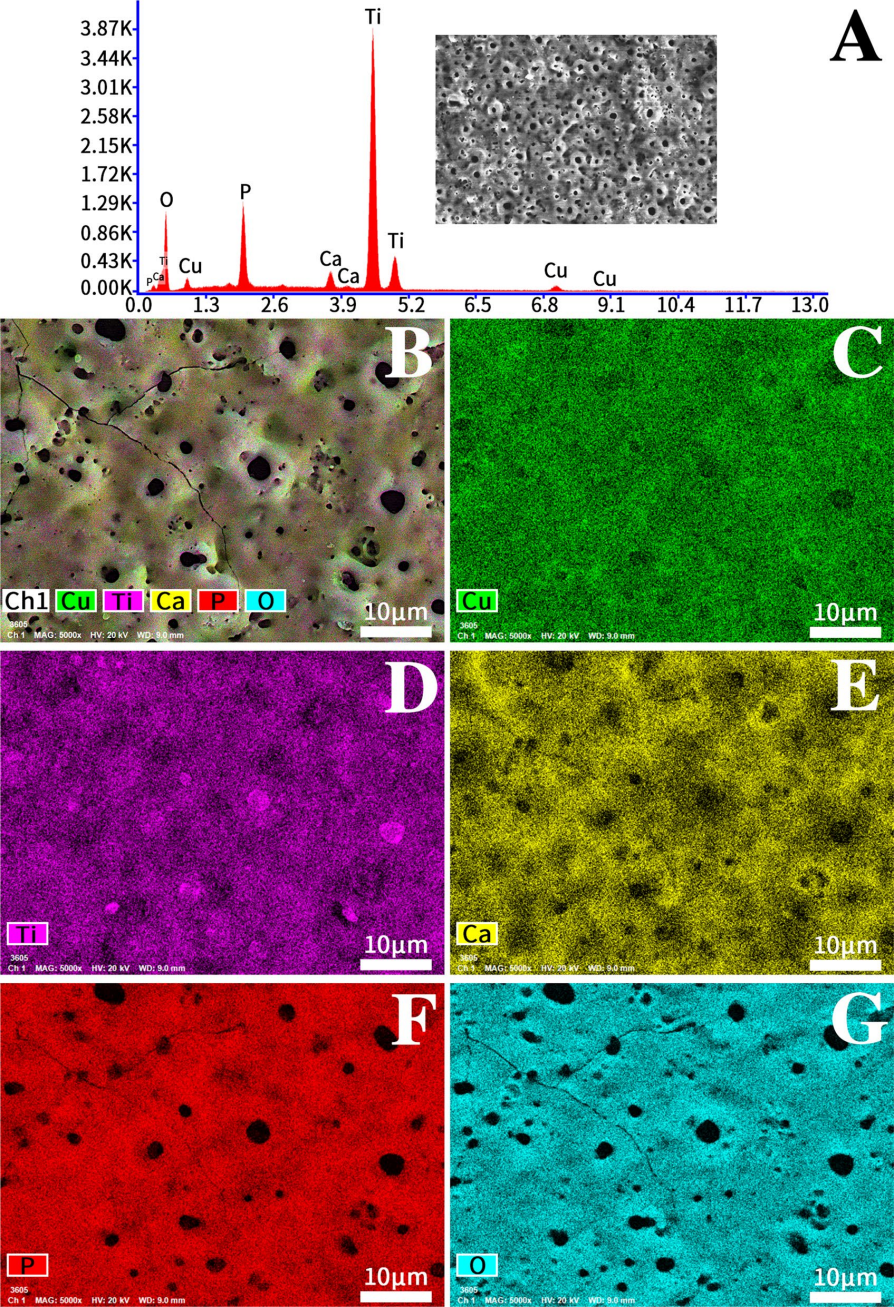
Mapping and EDS diagram of the Cu–TiO_2_ coating (TCP Cu II)

Figure 2 shows the mapping and EDS diagram of the microporous Cu–TiO_2_ coating As seen in the EDS diagram, the microporous Cu–TiO_2_ coating is composed of Cu, Ti, Ca, P and O. The solution containing Cu, Ca, and P was fully incorporated into the coating. More importantly, we did not find other toxic and harmful elements. The mapping results of the microporous Cu–TiO_2_ coating show that copper, calcium and phosphorus are evenly distributed in the coating.

**Fig. 3:**
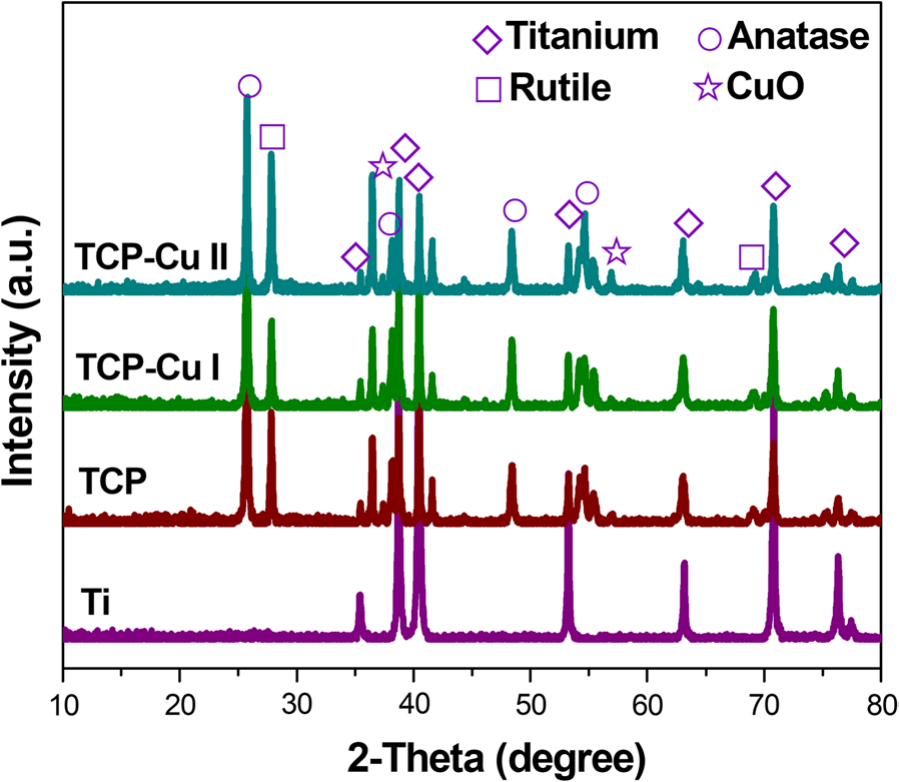
XRD patterns of Ti, TCP, TCP CuI and TCP CuII

Figure 3 shows the XRD patterns of Ti, TCP, TCP Cu I and TCP Cu II. All coatings are mainly Ti, rutile and anatase. More importantly, CuO appeared in the microporous Cu–TiO_2_ coating, which indicated that copper existed in the form of CuO.

**Fig. 4:**
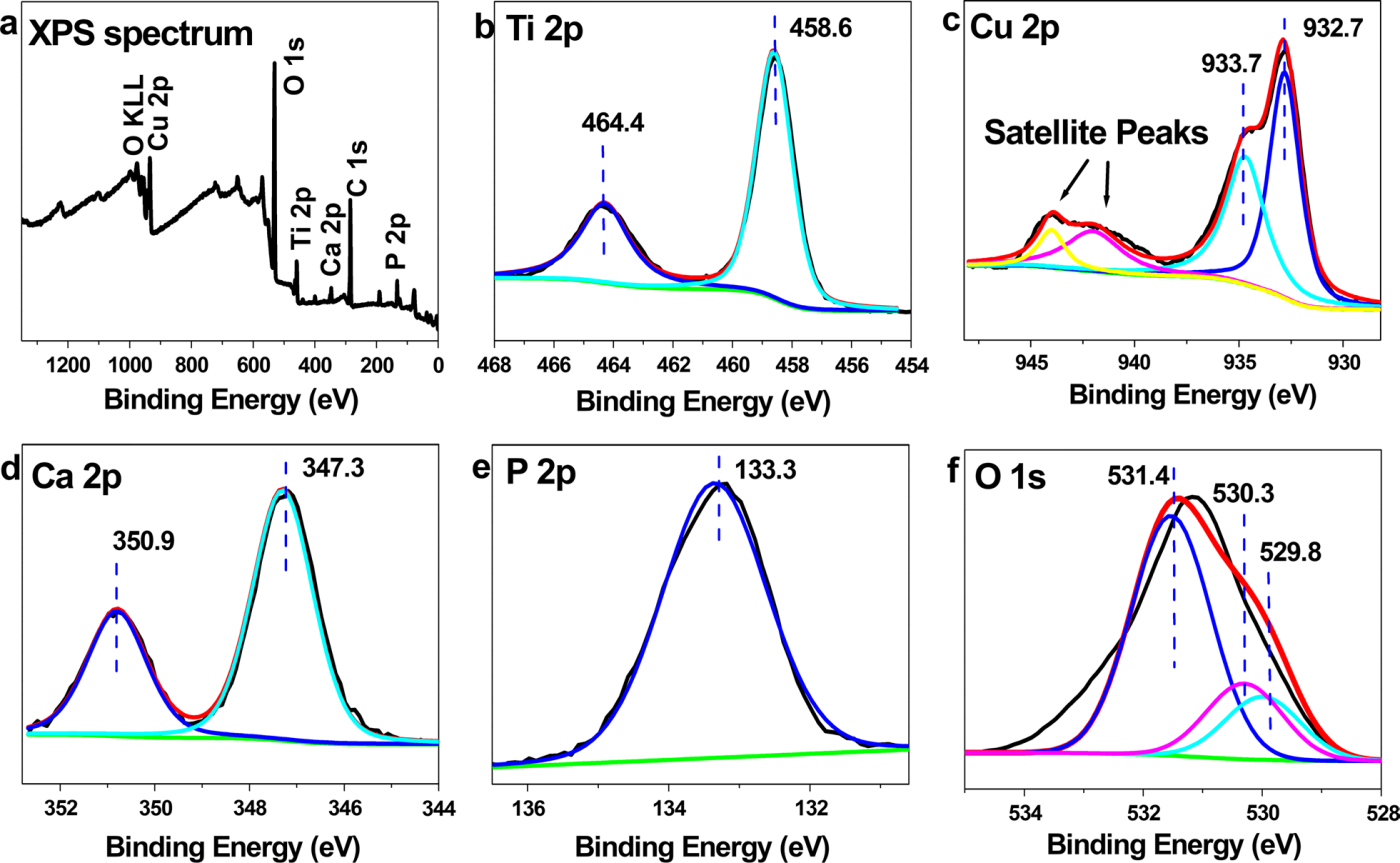
XPS image of the microporous Cu-TiO_2_ coating. **a** XPS spectrum, **b** Ti2*p*, **c** Cu2*p*, **d** Ca2*p*, **e** P2*p* and **f** O1*s* spectra

Figure 4 is the XPS image of the microporous Cu–TiO_2_ coating. Figure 3a shows the full spectrum of the microporous Cu–TiO_2_ coating determined by X-ray photoelectron spectroscopy, which is similar to the EDS result, except for titanium, oxygen, calcium and phosphorus. In addition to the characteristic peaks of copper, there are also characteristic peaks of copper. The peak in the Ti2*p* spectrum corresponds to TiO_2_, and the peak of Cu2*p* at 932.7 eV is considered indicative of CuO [17, 18].

**Fig. 5:**
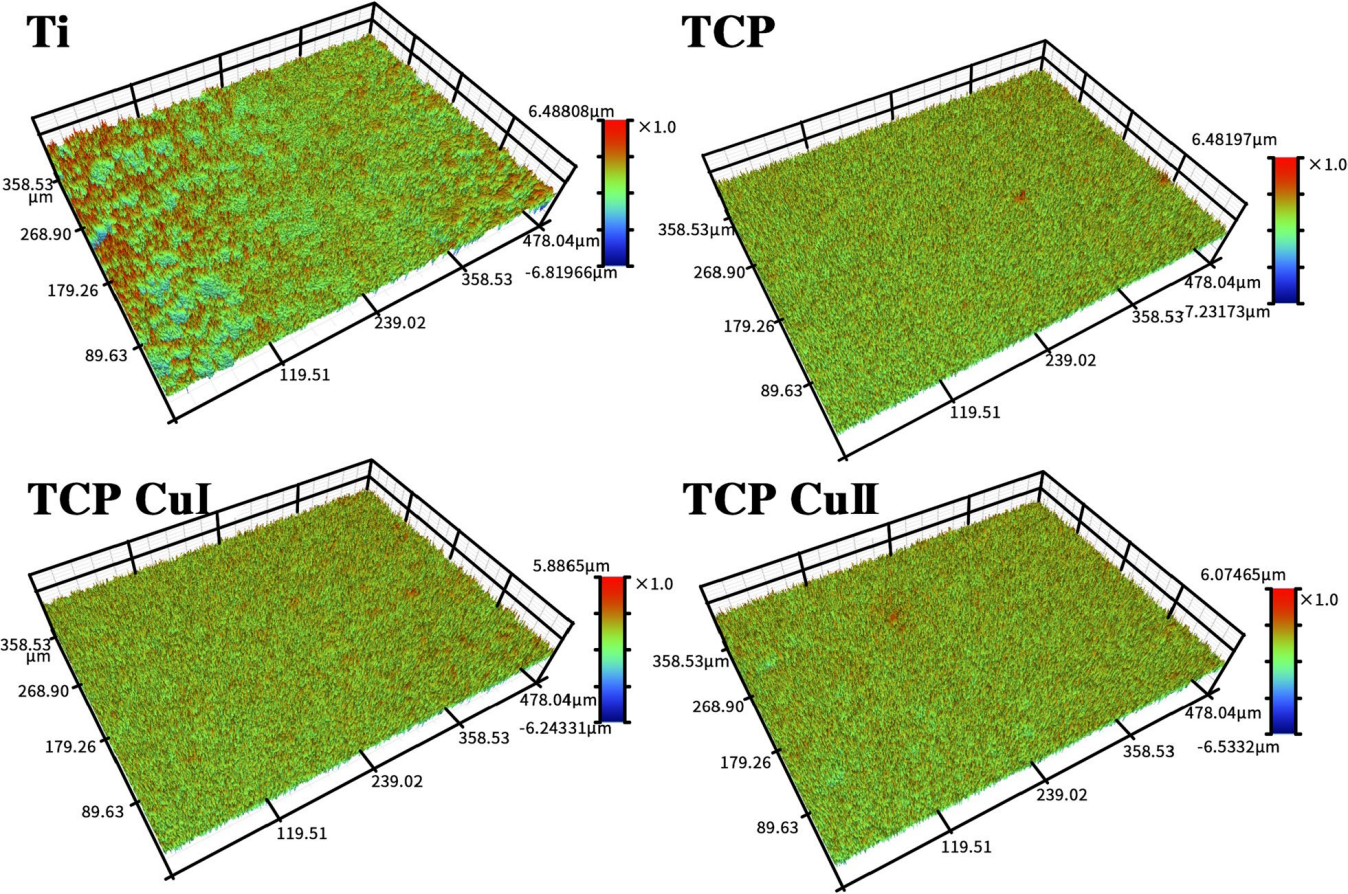
Profilometer morphology of different samples

Figure 5 shows the profilometer morphology of different samples. Except for the Ti samples, the profilometer surface morphology of each group is similar, which shows a volcanic-like multistage pore cavity structure. Further analysis of the roughness Ra of each group showed that the roughness of TCP, TCP CuI and TCP CuII was greater than that of Ti. The roughness of TCP, TCP CuI and TCP CuII is similar, and the difference is not significant, which indicates that microarc oxidation increases the roughness of Ti, but copper doping does not affect the roughness of the samples.

### Cell Adhesion and Proliferation

**Fig. 6:**
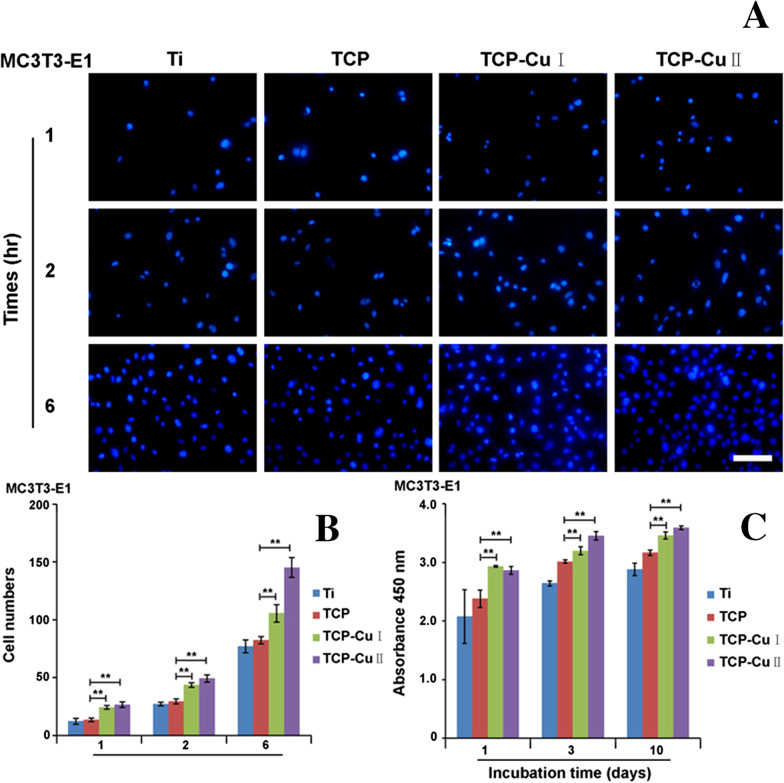
Adhesion and proliferation of MC3T3-E1 cells on the surfaces of different samples. **a** Cell adherent images at 1, 2 and 6 h after staining with DAPI, **b** bar chart of adherent cells and **c** bar chart of cell proliferation (Data are presented as mean ± SD, *n* = 5. **p < 0.01 compared with group TCP)

Figure 6a shows cell adherent images at 1, 2 and 6 h after staining with DAPI. Figure 6b the numbers of MC3T3-E1 cells adhered to the surfaces of different samples at different times. The numbers of adherent cells in different groups of samples at different time points are arranged in the following order: TCP Cu II > TCP Cu I > TCP > Ti. Compared with the Ti and TCP groups, the number of adherent cells in the TCP Cu I and TCP Cu II groups increased significantly. Therefore, the microporous Cu–TiO_2_ coating can significantly promote cell adhesion.

Figure 6c shows the proliferation of MC3T3-E1 cells on the surface of different samples at different times. Similar to the adhesion trend of the above cells, the cell proliferation of the TCP Cu I and TCP Cu II groups was significantly higher than that of the Ti and TCP groups. The surface morphology of the microporous Cu–TiO_2_ coating and copper ions together promoted cell proliferation.

**Fig. 7:**
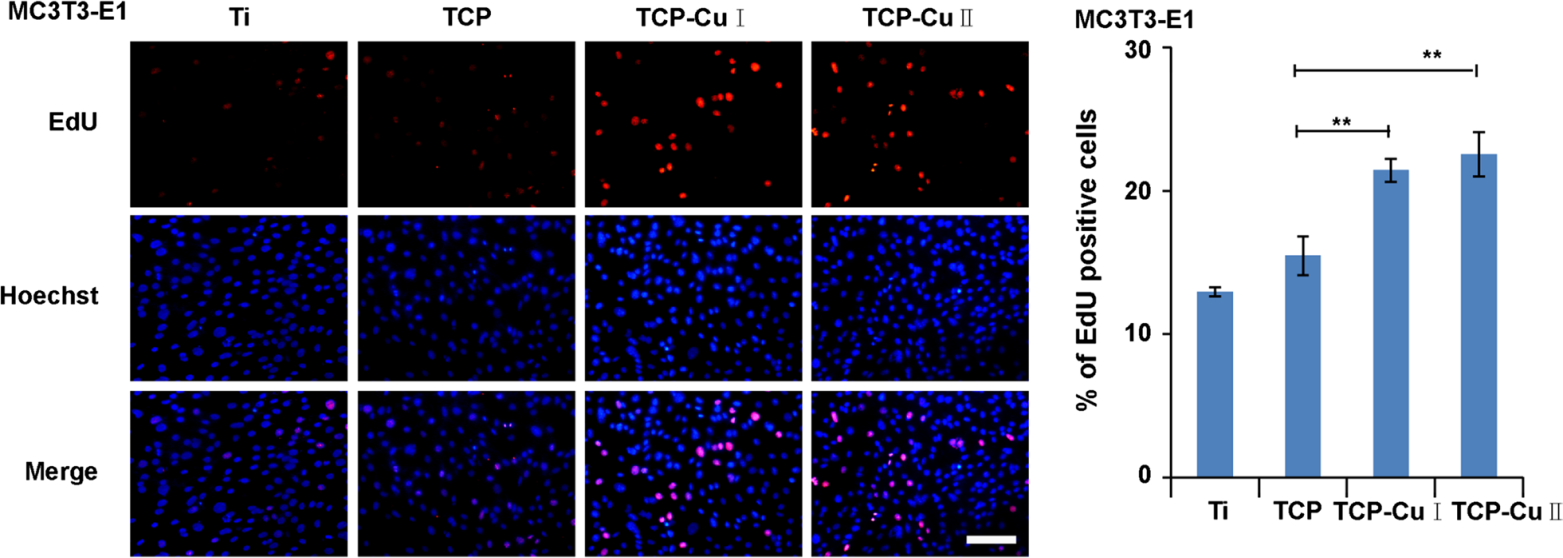
EdU staining measured after 3 days of culturing (Data are presented as mean ± SD, *n* = 5. **p < 0.01 compared with group TCP)

Figure 7 shows the EdU staining results. The ratio of EdU-positive nuclei followed this order: TCP CuII > TCP CuI > TCP > Ti. In comparison with the Ti group, the proliferation of cells in the TCP CuII group significantly differed.

### Live/Dead Staining

**Fig. 8:**
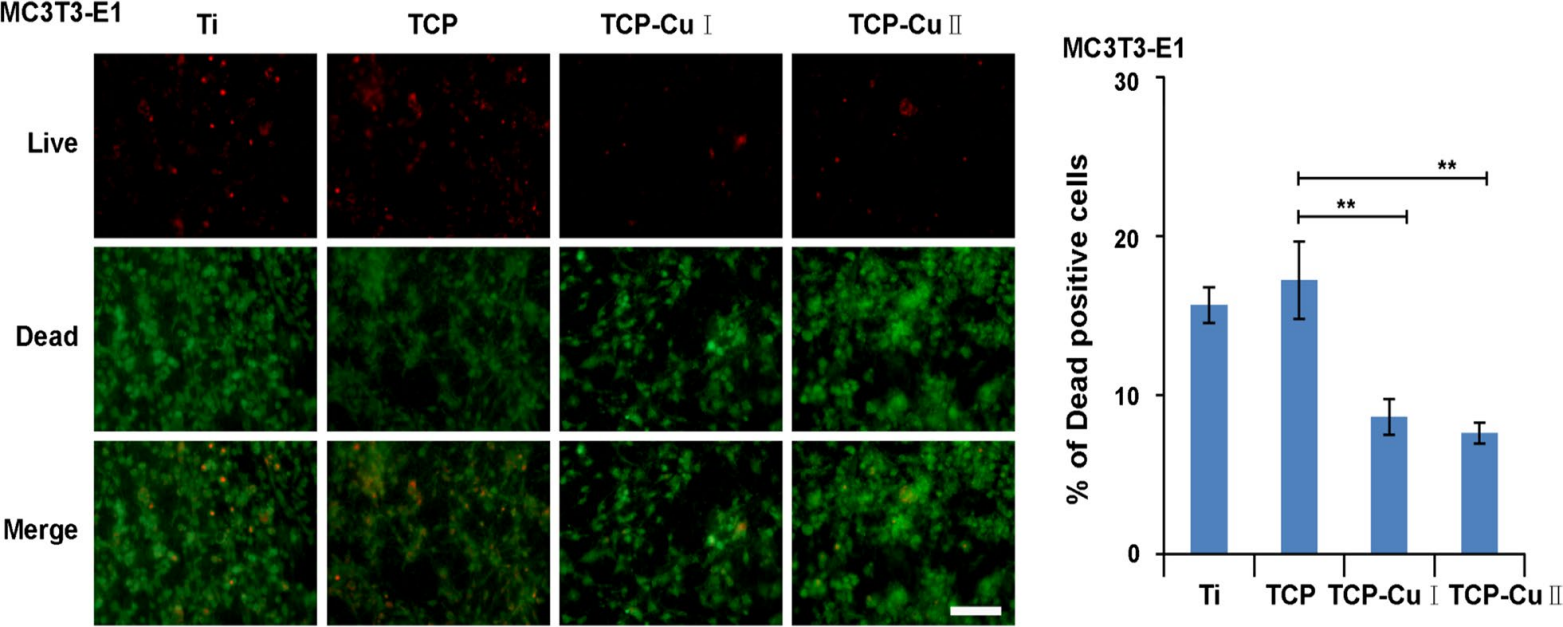
Staining of living/dead cells on the surfaces of different samples (Data are presented as mean ± SD, *n* = 5. **p < 0.01 compared with group TCP)

Cytocompatibility is the basic requirement of implant materials. Live/dead fluorescent staining can evaluate the cytotoxicity and biocompatibility of materials. Figure 8 shows the live/dead cell staining results after cells were cultured on the surface of different samples for 3 days. There were only a few dead cells (red) on the surface of each group of samples, indicating no obvious cytotoxicity. Copper doping in the microporous Cu–TiO_2_ coating does not obviously increase cytotoxicity, and it has good cell compatibility.

### Expression of Osteogenic Differentiation Genes

**Fig. 9:**
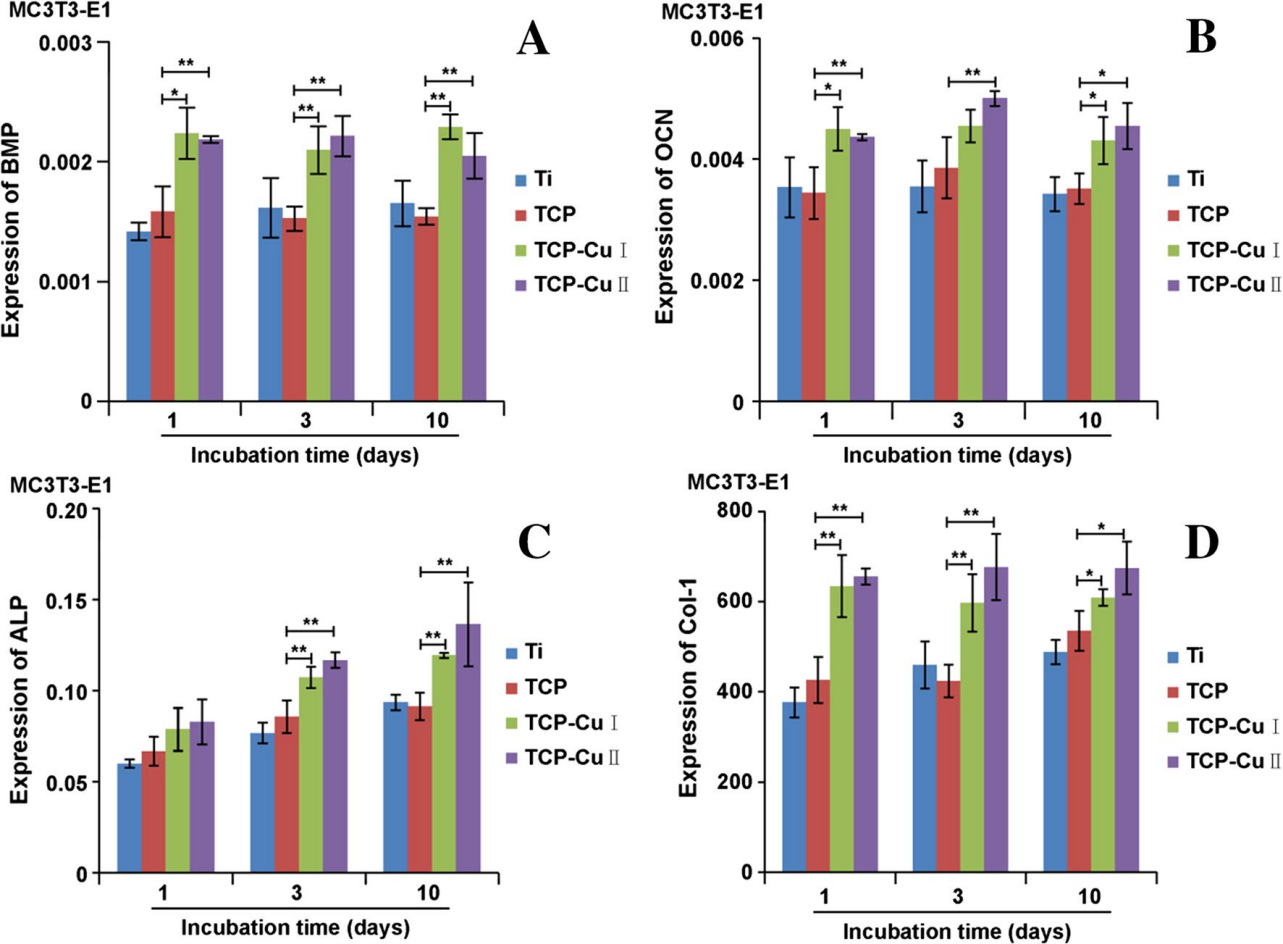
The mRNA expression of *BMP*, *OCN*, *ALP* and *COL-I* after 1, 3 and 10 days of incubation (Data are presented as mean ± SD, *n* = 5. *p < 0.05 compared with group TCP, **p < 0.01 compared with group TCP)

Figure 9 shows the mRNA expression levels of osteogenic differentiation genes (*BMP*, *OCN*, *ALP* and *COL-I*) on the surface cells of each group of samples at different time points. Over time, the expression of osteogenic differentiation genes on the surface of each group of samples gradually increased. At the same time, the expression of each group of genes showed the following trend: TCP Cu II > TCP Cu I > TCP > Ti. Compared with the Ti and TCP groups, the expression of bone-related differentiation genes composed of TCP Cu I and TCP Cu II was significantly enhanced, indicating that the microporous Cu–TiO_2_ coating can promote osteogenic differentiation.

### Gross Observation and Micro-CT Analysis

**Fig. 10:**
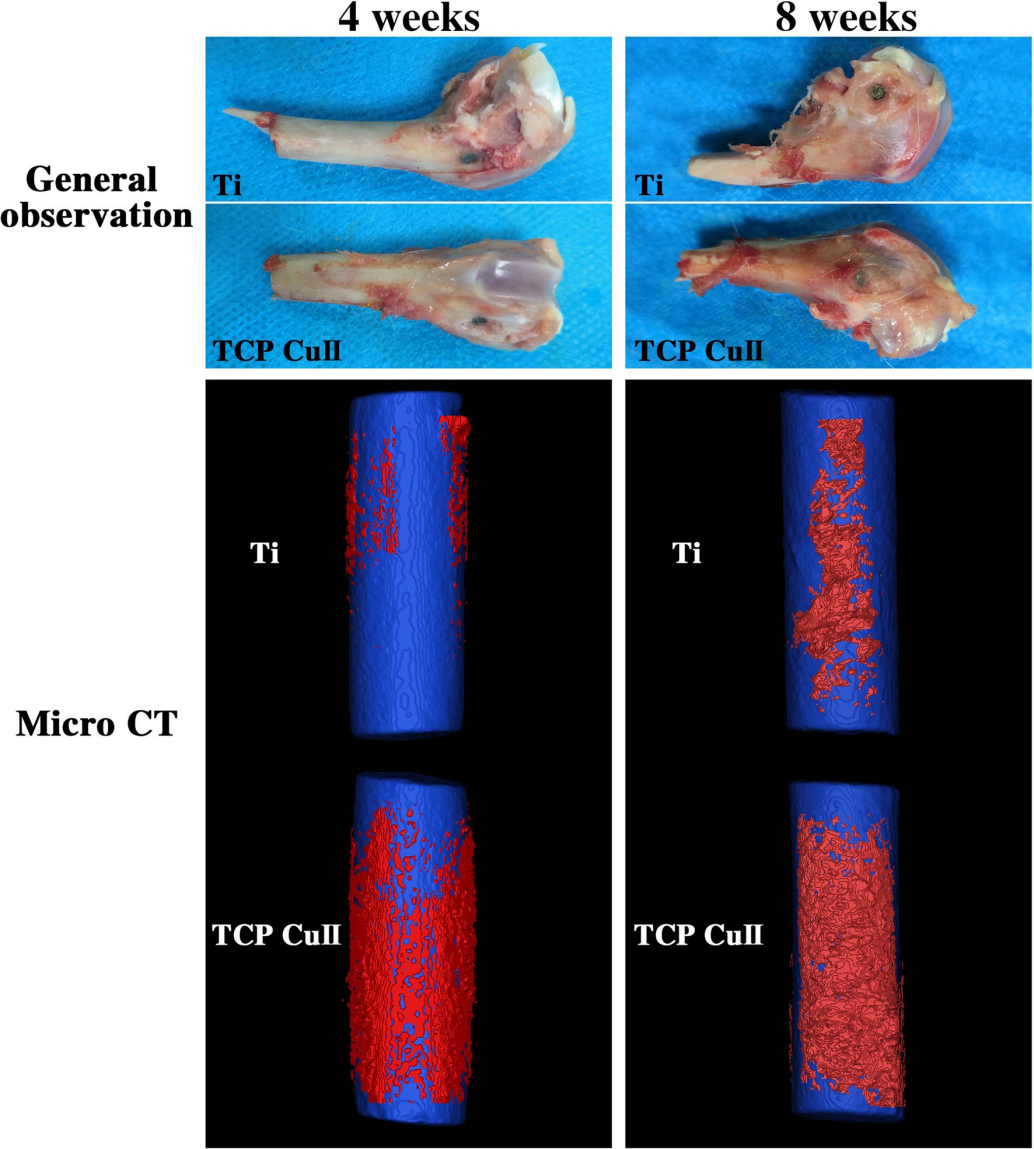
Gross observation and micro-CT reconstruction of the femoral condyle were observed at 4 and 8 weeks after implantation

Figure 10 shows the results of gross observation and micro-CT reconstruction of the femoral condyle. Gross observation showed that the implants in the two groups were in the middle of the femoral condyle at 4 and 8 weeks with good positioning, no obvious infection and no implant loosening. Micro-CT three-dimensional reconstruction shows that over time, the new bone tissue on the surface of the two groups of samples at 8 weeks was greater than that at 4 weeks, and at different time points, new bone tissue was formed on the surface of the microporous Cu–TiO_2_-coated titanium implant, and the amount was greater than that of the control group. By comparing the bone volume fraction of the two groups, the bone volume fraction (BV/TV) of microporous Cu–TiO_2_-coated titanium was significantly higher than that of the blank control group. Microporous Cu–TiO_2_-coated titanium can promote the osseointegration of titanium implants.

### Histological Evaluation

**Fig. 11:**
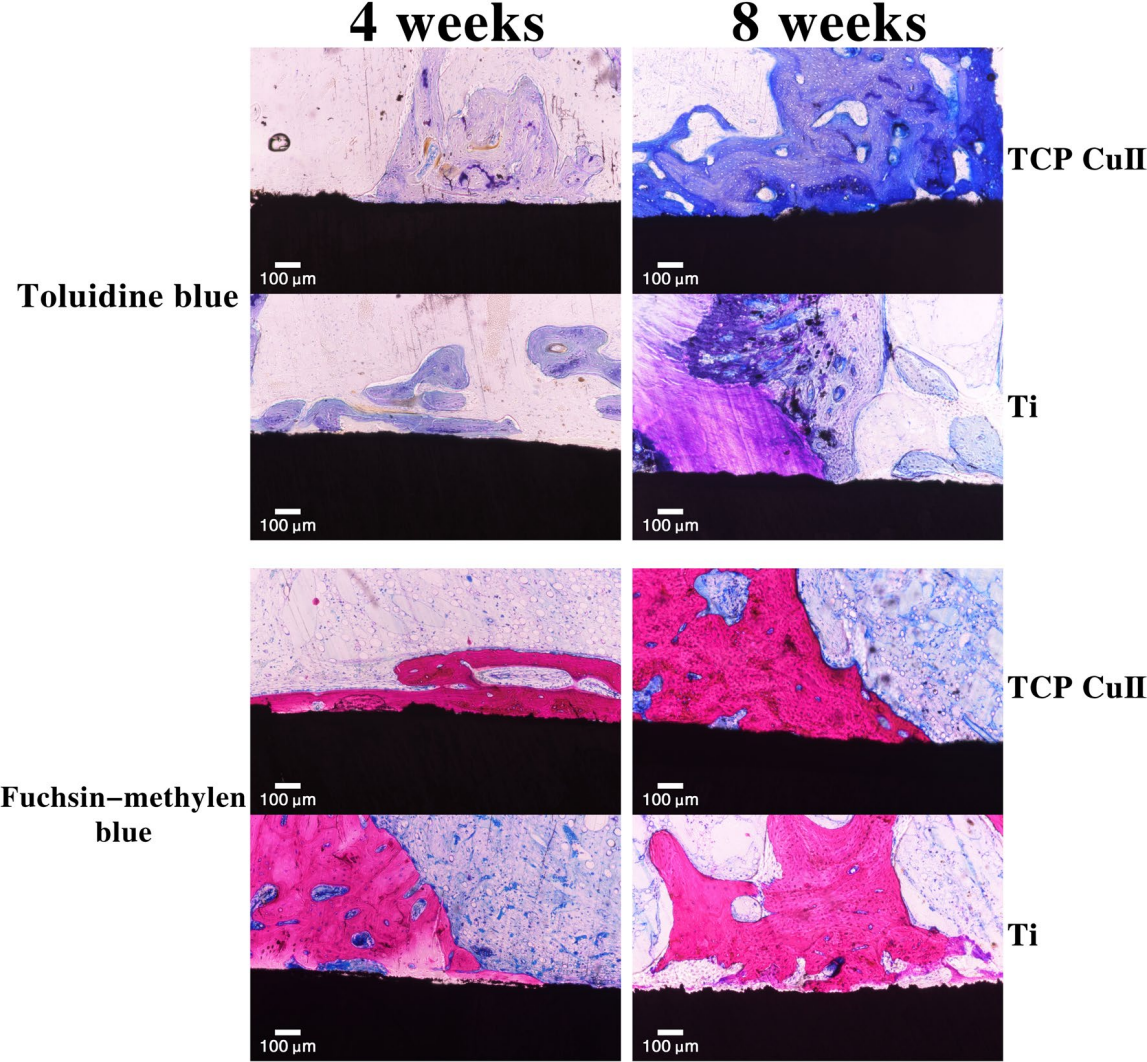
Toluidine blue and fuchsin-methylene blue staining of new bone formation at 4 and 8 weeks after implantation

Figure 11 shows the results of toluidine blue and fuchsin-methylene blue staining. No fiber envelope was seen at the bone-implant interface, indicating that the titanium implant had no inflammatory reaction at the interface with the bone. White gaps can be seen at the titanium implant-bone interface gaps in the two groups. The width of the white gaps in the control group was larger than that of microporous Cu–TiO_2_ coated titanium. The wider the gap, the weaker the induction of new bone tissue by the implants. Toluidine blue staining shows the blue band at the implant-bone interface gap, which is the new bone. Microporous Cu–TiO_2_ coated titanium had more bone tissue than the control group, indicating that microporous Cu–TiO_2_ coating can better promote osteogenesis and has a better osseointegration effect. The bone matrix around the microporous Cu–TiO_2_ coated titanium was thicker and continuous, and the bone tissue was significantly increased. In contrast, the control group had less bone. This result shows that microporous Cu–TiO_2_ coated titanium can better promote osteogenesis and has a better osseointegration effect.

## Discussion

Metal titanium and its alloys are widely used in dentistry, plastic surgery and other fields due to their excellent mechanical properties and biocompatibility; however, titanium as an implant can only passively combine with bone tissue. This combination is often a mechanical combination, which is prone to loosening and sinking of the implant, leading to implant failure. At present, the method of implant surface modification is mainly used to improve its osseointegration ability [19]. The surface of the ideal implant should have both osteoconductivity and osteoinductivity, good biocompatibility, and promote the formation of osseointegration between the implant and the bone tissue [20]. In this study, we prepared an innovative microporous Cu–TiO_2_ coating on the surface of titanium, hoping to improve the biological activity and biocompatibility of titanium and overcome the shortcomings of titanium implants in current clinical applications.

Copper ion and titanium dioxide have been proved to have good biological activity [21]. In this study, the microporous Cu–TiO_2_ coating prepared by microarc oxidation on the titanium surface showed the greatest advantage of tight bonding between the coating and the titanium substrate, which has been confirmed in the literature [22]. The good bonding force of the microarc oxidation coating is closely related to the formation process. In the process of microarc oxidation, chemical oxidation, electrochemical oxidation, and plasma oxidation coexist. Under the action of the instantaneous high temperature and high pressure generated by arc discharge, the titanium surface is grown on the surface of the substrate in a “growth” manner, mainly the substrate oxide. The ceramic coating, coating and substrate canine teeth are staggered, and they have a good bonding force [23].

The surface characteristics of biological materials directly affect the biological characteristics of the materials. The microarc oxidation coating presents a rough and porous surface morphology under an electron microscope. The morphology is mainly composed of micropores of different sizes that interpenetrate with each other. These small pores are formed in the process of microarc oxidation, the metal surface is broken down under high voltage, and the instant high-temperature sintering in the microarc zone directly oxidizes and sinters the titanium matrix into a ceramic film with a crystalline ceramic phase structure, where electrical breakdown occurs. The micropores observed under the electron microscope are formed. These rough and porous structures can not only increase the attachment area of tissue cells, but these interpenetrating micropores are also equivalent to a three-dimensional scaffold structure, which can induce bone tissue to grow into the pores and promote cell adhesion and extension. Pan et al. [24] prepared micro/nanohierarchical structured TiO_2_ coatings on polished titanium by micro-arc oxidation and found that the coatings were favorable for the adhesion and extension of MG63 cells. Zhang et al. [25] prepared a Si–TiO_2_ coating by micro-arc oxidation, and further study showed that the adhesion of MC3T3-E1 cells on this silicon-containing TiO_2_ coating was significantly higher than that on a Si-free TiO_2_ coating and pure Ti.

The greatest advantage of the microarc oxidation coating is that the ions in the electrolyte solution can be introduced into the coating during the microarc oxidation process. In this study, the EDS analysis results of the coating surface showed that the microporous Cu–TiO_2_ coating is mainly composed of Cu, Ca, P, O and Ti elements, of which titanium comes from the matrix, calcium and phosphorus come from the basic electrolyte solution, and the copper ions in the electrolyte are deposited in the coating along with the formation of the ceramic film. The calcium and phosphorus components on the surface of the implant can not only improve the surface properties of the material but also induce bone formation. In addition to calcium and phosphorus, the copper ions in the microporous Cu–TiO_2_ coating have good biocompatibility and biological activity. Copper-doped coatings on the surface of implants have also been reported in the literature. Astasov-Frauenhoffer et al. [26] deposited copper on Ti via a spark-assisted anodization method and confirmed that the viability of the bacterial cells was strongly inhibited. Zong et al. [27] combined anodization and magnetron sputtering to combine copper into TiO_2_ nanotubes and prepare copper (Cu) into TiO_2_ NTAs (Cu–Ti–O NTAs), and further study showed that Cu–Ti–O NTAs have excellent long-term antibacterial ability and favorable angiogenic activity.

Biocompatibility is the minimum requirement for measuring implants and is also the basic guarantee for implant safety. In this study, biologically active copper was introduced into the surface of titanium implants through microarc oxidation; however, copper ions, as heavy metal ions, have potential toxicity. Therefore, we must consider whether the microporous Cu–TiO_2_ coating is cytotoxic. In this study, live/dead cell staining was used to evaluate the microporous Cu–TiO_2_ coating. The results showed no obvious cytotoxicity on the surface of the microporous Cu–TiO_2_ coating on the titanium surface, and good cell compatibility was observed. We speculate that this finding may be related to the low copper content of the coating. Huang et al. [28] fabricated gap-bridging chitosan–gelatin nanocomposite coatings incorporated with different amounts of copper (Cu; 0.01, 0.1, 1, and 10 mM for Cu I, II, III, and IV groups, respectively) on Ti and demonstrated that the activities of bone marrow stromal cells were not impaired on Cu-doped coatings except for the Cu IV group.

Cell adhesion and proliferation are the basis of implant osseointegration in the later stage. The more cells that adhere and proliferate on the surface of the implant, the better the effect of implant-bone interface osseointegration. The results of this study showed that on the first day after the material surface was inoculated, the amount of cell adhesion on the surface of the samples of each group differed, and the amount of adhesion on the surface of the microporous Cu–TiO_2_ coating group increased significantly. The number of cells that adhered to the sample surface gradually increased, but the number of cells that adhered to the group with microporous Cu–TiO_2_ coating was significantly greater than that of the other two groups. The difference was statistically significant, indicating that the microporous Cu–TiO_2_ coating was doped with copper ions. A porous, rough surface is most conducive to cell adhesion. Similar to cell adhesion, cell proliferation on the surface of each group of materials also showed similar results. Our research results are similar to previous reports [29].

In addition to adhesion and proliferation, the degree of cell differentiation on the surface of the material can further reflect the performance of the implant's osseointegration. The osteogenic differentiation marker genes *ALP*, *BMP*, *RUNX2*, *OCN* and *COL-I* can reflect cell differentiation. In this study, as time went by, the expression of *BMP, OCN, ALP* and *COL-I* on the surface of each group of samples increased, but the expression of the microporous Cu–TiO_2_ coating group was significantly higher than that of the control group. This finding is closely related to the promotion of osteogenic differentiation by copper ions. Komarova et al. [30] prepared Zn- and Cu-containing CaP-based coatings by microarc oxidation on Ti and showed that low amounts of Cu and Zn in the coatings promoted high motility of human adipose-derived multipotent mesenchymal stromal cells and subsequent ability to differentiate into osteoblasts.

Osseointegration is the key to the success or failure of the implant. This means that there is no fibrous tissue between the implant and the bone tissue. There is direct contact between the implant and the bone tissue, and it can directly bear stress to realize the relationship between the implant and the bone tissue, establishing a functional connection. The osseointegration between orthopedic implants and bone tissue is affected by many factors, such as the initial stability of the implant and the mechanical properties of the implant material, implant surface properties, biocompatibility, biological activity and the condition of the surrounding bone tissue [31].

An ideal implant position and a stable biomechanical environment are the prerequisite and basis for the osseointegration of the implant-bone interface. In this study, the femoral condyle was chosen to be implanted with a copper-doped microporous coating because the abundant blood supply and sufficient bone volume at the femoral condyle can provide a good anatomical basis and a relatively stable mechanical environment for the implant. Moreover, the femoral condyle is mostly cancellous bone. After implantation, bone formation and the effect of implant-bone interface osseointegration can be more intuitively evaluated.

Micro-CT is currently a common method for observing the osteogenesis performance of implants and is also an effective method for evaluating the osseointegration between implants and bone tissue. Bone microstructure is visualized through three-dimensional reconstruction and the region of interest (ROI) analysis with the assistance of related software to obtain the relevant parameters of new bone tissue. Among all the parameters, BV/TV represents the total amount of bone formation and is an important indicator reflecting the osseointegration of the implant. In this study, we chose BV/TV as the detection index. Four weeks after implantation, the BV/TV of the microporous Cu–TiO_2_ coating group was higher than that of the control group. Eight weeks after implantation, the BV/TV values of the microporous Cu–TiO_2_ coating group and the control group were higher than those at 4 weeks, and the BV/TV of the microporous Cu–TiO_2_ coating group was higher than that of the control group. On the basis of micro-CT detection, we performed histological observation and quantitative analysis of the bone tissue around the implant through hard tissue slices. The results of VG staining showed that the microporous Cu–TiO_2_ coating group formed more new bone than the control group, and the new bone that formed around it was in direct contact with the internal implant without fibrous tissue infiltration. These results indicate that microporous Cu–TiO_2_ coating on the titanium surface can promote the osseointegration of titanium implants. This finding is similar to previous in vitro studies. The rough, porous structure produced by microarc oxidation mimics the micro/nanostructure of normal bone tissue. More importantly, biologically active copper ions promote bone tissue regeneration. Under the action of these common factors, bone tissue regeneration on the surface of the implant is promoted. Our research results are consistent with literature reports. Milan et al. [32] designed a multifunctional Cu/a-C:H thin coating deposited on Ti–6Al–4 V alloy (TC4) via magnetron sputtering and found that the coating composition can stimulate angiogenesis and osteogenesis and control the host response, thereby increasing the success rate of implants.

## Conclusion

In summary, we prepared a microporous Cu–TiO_2_ coating on a titanium surface by microarc oxidation. The surface of the coating has a porous structure with pores of different sizes and interconnected pores. The coating increases the surface roughness of Ti and copper is evenly distributed on the surface of the coating. In vitro studies revealed that the coating has no obvious cytotoxicity and can promote the adhesion, proliferation and differentiation of MC3T3-E1 cells. In vivo experiments further confirmed that the coating can induce the formation of new bone tissue and promote osseointegration at the titanium implant-bone interface. In view of the biological activity in vivo and in vitro, we believe that the microporous Cu–TiO_2_ coating on the surface of titanium implants has potential clinical application value in orthopedics.

## Data Availability

Not applicable.

## Abbreviations

MAO: micro-arc oxidation
Cu: copper
Zn: zinc
Cu–TiO_2_ coating: copper–titanium dioxide coating
ROI: region of interest
ALP: alkaline phosphatase
